# New Machine, Old Cough: Technology and Tuberculosis in Patna

**DOI:** 10.3389/fsoc.2020.00018

**Published:** 2020-04-03

**Authors:** Vaibhav Saria

**Affiliations:** Department of Gender, Sexuality, and Women's Studies, Simon Fraser University, Burnaby, BC, Canada

**Keywords:** tuberculosis, technology, expertise, diagnosis, India, anthropology

## Abstract

In 2013, a new technology, GeneXpert, was introduced in India, which, in addition to testing for TB, could also diagnose whether the detected strain was drug resistant. By detecting the bacterium more effectively than other available tests and simultaneously testing for resistance, GeneXpert promised to reduce the delay in diagnosis and hence ineffective treatments. The new test was introduced to multiple cities via a coalition that included global health funding bodies, the government of India, the World Health Organization, and non-governmental organizations. Despite the concerted effort of the coalition, among formal providers (those trained in biomedicine) in the private sector, the new technology was not adopted as quickly as had been hoped. Examining formal providers' initial responses to the technology's introduction in the city of Patna reveals how the adoption of new technology can be influenced by the particularities of the local medical market such as the availability of diagnostic tests, presence of informal providers, and reputation of formal providers. While protocols and operations might seem standardized across implementation plans, the work that is required to ensure success must take into account the particular role that the market plays from site to site.

## Introduction

During the past decade, a new technology became available for diagnosing tuberculosis (TB) in India that seemingly remedied the inefficiencies of the existing technology, namely, the sputum smear microscopy (or the sputum Acid Fast Bacilli)^1^. In 2010, the automated Cartridge-Based Nucleic Acid Amplification Test (CBNAAT) was endorsed by the World Health Organization (WHO) and subsequently promoted by India's National Strategic Plan (NSP) for Tuberculosis Control for the years 2012–2017 [World Health Organization (WHO), 2013]. This diagnostic test not only delivers results in 2 h but also shows whether the TB bacterium has developed resistance to rifampicin, a drug widely used as the first-line treatment for TB. During this period, in the eastern Indian city of Patna, a network of actors used a new model of global health intervention called the Private Provider Interface Agency (PPIA) to introduce the CBNAAT product, branded and marketed as GeneXpert (Gopalakrishnan, 2015). Health providers received the new technology, marketed with the promise and hope of solving the complex issue that is India's TB epidemic, with suspicion. I conducted over 20 months of ethnographic research with various actors in the existing medical infrastructure in Patna as part of a team evaluating the impact of the PPIA health intervention. My ethnography included interviewing as well as shadowing formal health care providers trained in biomedicine, lab technicians, patients, compounders, and pharmacists. Wariness to new technology must be understood in relation to the history of a particular medical landscape. While the intervention is currently still in place, the ethnography reflects the initial concerns and anxieties of actors in the field that provoked innovation and modifications in implementation to ensure success.

GeneXpert did not simply replace older technologies such as chest X-rays or sputum Acid-Fast Bacilli smear tests (“sputum AFB”), the existing methods used by providers to diagnose patients with TB. The providers in the city had fine-tuned and built their own clinical protocol to diagnose patients with TB through decades of experience. They were unwilling to set their history of practice aside to passively follow the directions set forth by the new technology. Instead, they embarked on a critical assessment of the technology and absorbed it into their protocol for evaluating and diagnosing patients in varying ways. These myriad ways of using a new piece of technology not only reflect the heterogeneous protocols and definitions of health but also reveal the impact of intractable particularities of one site or city on the uptake or success of that technology. In other words, the promise of new medical technology depends on whether it enables or endangers the expertise and authority of the provider that is constitutive of being diagnosed with a disease.

## Old Disease, New Concerns

In the 1990's, the HIV epidemic along with the spread of drug-resistant TB spurred the Indian state to implement new measures to test, treat, and control the TB epidemic. The resulting plan, called the Revised National Tuberculosis Control Plan (RNTCP), centered around strategies of Directly Observed Treatment–Short Term, popularized through the acronym DOTS and its expanded version, DOTS-Plus. These strategies were piloted, implemented, and scaled up in phases that lasted from 1993 to 2005. The limitations and failures of these strategies, ranging from alternate day regimen, requiring that the medicine be taken in front of the clinic's staff, and conflating completion of treatment as per protocols with curing of TB, are well-documented (Das and Das, 2007; Ecks and Harper, 2013; Das, 2015). These failures have also shown to increase the vulnerability of patients to developing drug resistance rather than prevent them. One of the changes that DOTS aimed to achieve was a reduction in the use of X-rays to diagnose TB and concurrently to encourage the use of sputum AFB. Variations in clinical presentation, inciting and reporting clinical histories, and differential diagnoses made X-rays a poor choice for diagnosing a patient with TB because they lacked disease specificity. Since the DOTS centers were based in the public sector, it was possible for the program to achieve the high targets set for diagnoses based on sputum AFB and completed treatment rate [World Health Organization (WHO), 2000]. Yet, the TB epidemic failed to come under control and the risk of drug-resistant TB increased. One of the important factors that emerged as crucial to combating TB was the large number of patients who continued to get diagnosed and seek treatment in the private sector, whose estimated proportion of TB patients was large but not precise (Pathania et al., 1997).

While suspicions on the part of the state regarding the quality and cost of care in the unregulated private sector that resulted in patients not completing or defaulting on treatment were well-founded based on existing research, efforts to engage the private sector were limited to a set of pilots and experiments (Dewan et al., 2006; Satyanarayana et al., 2011). At the end of the second phase (2005–2012) of RNTCP, it was evident that the private sector would have to be tackled at multiple levels for India to rid itself of TB. Since TB was not declared a notifiable disease until 2012 under the Epidemic Diseases Act of 1897, there was no way of knowing how many patients with TB were diagnosed or treated in the private sector (Bhaumik and Biswas, 2012; Rakesh, 2016). It was estimated that at least a million cases were “missing” in India—“missing” signifying either the patients had been diagnosed but not notified or not diagnosed at all [World Health Organization (WHO), 2014a; Raizada et al., 2015]. To capture the “missing million,” efforts to engage the private sector were renewed in the hope that providers would not only notify patients they had diagnosed but also change their behavior to test patients for TB sooner rather than later. It was expected that the new technology of GeneXpert that was introduced by the PPIA intervention would facilitate this change in behavior.

## Time, Triage, and the Private Sector

The imperative to engage the private sector would not have emerged if the “missing million” were being treated adequately: if patients were receiving adequate treatment, it would be reflected in a substantial reduction in mortality from TB. The inadequacy and hence the critical importance of engaging the private sector centered on the issues of misdiagnosis and delay in diagnosis. Several sets of research and data were pieced together by Global Health researchers to create a linear narrative of the patient's journey of being diagnosed in the private sector, which then informed the strategy of the PPIA. After the second phase of the RNTCP (2005–2012), the Central TB Division of the Indian government drafted a National Strategic Plan (NSP) that was to last from (Central TB Division, Directorate General of Health Services, Ministry of Health and Family Welfare, 2012-2017). The NSP cited the National Family Health Survey that was released in 2005–2006 that showed that a provider in the private sector was the first point of contact for more than 80% of all patients. The NSP explained that patients were switching from private to public providers as a result of economic pressures that grew higher because of the long duration of TB treatment, ineffective treatment for their illness, and a lack of follow-up that ensured completed treatment and hence exacerbated the risk of developing drug resistance (Central TB Division, Directorate General of Health Services, Ministry of Health and Family Welfare, 2012, p. 30). The “missing million” were framed as patients facing a delay in proper diagnosis, a delay in treatment, or incomplete and partial treatment resulting in a higher risk of mortality. Furthermore, with the established risk of acquiring drug-resistant TB infection directly rather than only through intermittent and improper use of the anti-TB regimen, it became even more urgent that the private sector be engaged in order to shorten the delay in diagnosing TB patients. Shortening that delay emerged as a focal point, one that would guide interventions to correct the multiple issues that defined the persistent epidemic, and GeneXpert was thought to be the “solution in a box” that would remedy misdiagnoses, detect drug resistance, and consequently reduce delay (Redfield, 2012).

A systematic review of existing literature in public health was conducted, which calculated that the median delay from onset of symptoms to diagnosis and treatment was 55.3 days (Sreeramareddy et al., 2014). Furthermore, the authors of this review calculated that patients consulted an average of 2.7 health care providers, including those called informal or unqualified providers—or more pejoratively, “quacks”—before patients were correctly diagnosed with TB. The vast private medical sector in India includes private hospitals and physicians with medical degrees trained in biomedicine who practice privately in clinics of varying sizes as well as a heterogeneous mix of informal providers. Das (2015) offers a concise view of how varied the informal sector is: it can be divided not only according to practitioners' training and discipline (Ayurveda, Yoga, Unani, Siddha, Homoepathy, and Naturopathy) but also according to the legitimacy offered to them by the state. Since many of these disciplines have a continuous history of negotiation with the state regarding the legality of their practice, informal providers might or might not have formal training, licenses, and certificates. Alongside these providers are also informal providers who offer services that range from administering injections, dispensing medicines, to running clinics and nursing homes that provide pregnancy and childbirth care, emergency care, and other services. These providers once again can be sanctioned by the state to practice through various licenses that certify them as Rural Medical Practitioners or Private Medical Practitioners to fill the gap left by the lack of state provision in the rural areas (Das, 2015, p. 159–180).

Anthropological studies looking into why the informal sector cannot simply be ignored, avoided, or policed show how these bodies of medical knowledge and practice enjoy a robust life in India at multiple levels, including the neighborhood, the rural, and the national (Langford, 2002; Barrett, 2008; Lambert, 2012; Ecks, 2013; Alter, 2014). Yet, the informal sector is mostly configured in public health as the source of problems, including the problems of delayed diagnosis of TB and drug resistance—both of drug-resistant TB and of antibiotic resistance—and therefore must be heavily policed (Satyanarayana et al., 2016). Harper writes, “A DOTS program officer in Uttar Pradesh assumed that 60–70 per cent of resistance was due to “barefoot doctors” or quacks and the misuse of rifampicin by them, and the giving of incorrect combinations” (Harper, 2009, p. 54). Even in studies that attempt to map the TB patient's journey from symptom to diagnosis through surveys and interviews, the informal provider emerges as a necessary target for intervention in attempts to address delay in diagnosis, despite clues pointing to complicated relationships between poverty, illness, everyday life, expertise, and technology (Uplekar et al., 2001; Kapoor et al., 2012; Wells et al., 2015; Mistry et al., 2016).

While part of the blame for missing or wrongly diagnosing patients was put on the informal providers who did have the expertise, another part was placed on outdated or faulty technology that made it difficult for formal providers to use their expertise to detect TB cases. The cheapest and most accurate test for an active TB infection for the last 125 years has been the sputum AFB, which tests the presence of the bacteria by staining the sputum sample with a reagent. Given the notorious lack of specificity and sensitivity of other tests such as X-rays, serological tests (TB-Gold), and tuberculin skin tests (the Mantoux test), the renewed efforts to control TB since the 1990's have emphasized the use of the sputum AFB test and set high targets for the number of TB cases to be confirmed through sputum microscopy (Pio et al., 1997). There were several proximate reasons for this insistence on sputum AFB, and they emerge from what Lock and Nguyen have called “biosocial differentiation” (Lock and Nguyen, 2010) referring to “the continual interactions of biological and social processes across time and space that eventually sediment into local biologies” (Lock and Nguyen, 2010, p. 90). Local biologies in the form of comorbidities and reinfections made providers think of differential diagnoses and consequently made X-rays unreliable. The older technology of the Mantoux test that still exists has been proven to be effective only in measuring latent TB infection, which once again is not helpful for diagnosing an active TB infection in India, where given the well-documented history of widespread TB infection among the population, a majority of that population, if not all, have latent TB (Little et al., 2015). Furthermore, the serological tests that were banned in 2012 because of their inaccuracy remain popular in Patna. The PPIA intervention incentivized informal providers to direct patients with the classic symptoms of TB to formal providers because it would correct one point in the cascade of care that purportedly caused the delay of diagnosis and the formal providers were incentivized to encourage the uptake of GeneXpert. It was assumed that formal providers, as rational actors, would quickly realize the benefit of a quick diagnosis that the technology offered and would then use it on their own even without incentives.

## The Private Provider Interface Agency

During the second phase of the RNTCP (2005–2012), efforts to engage the formal providers in the private sector were renewed and schemes were implemented that would allow formal providers to test patients for free. To achieve the twin goals of ensuring patients were diagnosed on the basis of sputum AFB as well as ensuring that the standardized treatment for TB was provided, the state activated an extensive infrastructure to direct private providers and their patients to the public sector. Grants were made available to non-governmental organizations (NGOs), community-based organizations (CBOs), and private providers in order to assist and facilitate the direction of the patients from the private sector to the public sector, alongside engaging private diagnostic labs where sputum samples could be tested. After judging the technical capacity and quality of the labs, RNTCP allowed these labs to be known as Designated Microscopy Centers, where the private providers could send their patients. In other words, it could be said that these private labs served as proxies for government labs, but only for TB patients. Among private labs, 12,000 were designated. In places where such labs were not available, sputum collection centers were set up so that they could be then transported to the Designated Microscopy Centers or to the labs in the public sector. Furthermore, the NGOs, CBOs, and private providers who agreed to partner for such schemes were given grants to hire staff to transport the sputum samples from clinics to the labs (Central TB Division, Directorate General of Health Services, Ministry of Health and Family Welfare, 2008). The failure of this vast infrastructure activated by the public sector further strengthened the argument to engage the private sector.

Several changes entirely reconfigured the engagement between the public and the private sector with respect to the TB epidemic. The most noticeable change was effected by making TB a notifiable disease in 2012 because while previously referral implied handing over the patients to the public sector, legally required notification meant that while the private sector could continue testing and treating patients, the state would have a larger role in supervising and implementing standardized protocols in the private sector. Because the previous objectives of limiting the private sector's share of diagnosing and treating TB patients failed, the new goal of engaging the private sector also meant that while the state could require private providers to notify, it could not make it mandatory for the private providers to direct patients to the public sector for care. The split between notifications and referral implied that the state and the private market had to negotiate the terms and conditions under which private providers were willing to be policed, disciplined, and punished for their management of TB patients. While the providers were required to notify and follow the official protocol outlined in the *Standards for TB Care in India*, they were allowed to make patients buy medicines privately, since free medications were only available in the public sector [World Health Organization (WHO), 2014b]. The language of policing is not surprisingly couched in terms of support offered rather than support needed: the guidelines state that “notification gives an opportunity *to support private sector* for following standardized practices in terms of Standard TB Care” (emphasis mine, Central TB Division, Directorate General of Health Services, Ministry of Health and Family Welfare, 2012, p. 4). This change in increasing state control is reflected in the guidelines for private providers where they are assured that the health worker will only check whether the patients have been treated according to the official protocol and offer “TB treatment under RNTCP, if desired by the patients” (Central TB Division, Directorate General of Health Services, Ministry of Health and Family Welfare, 2012, p. 8).

To gauge how such a change in the private sector could be effectively implemented, a new intervention was designed, the PPIA, and piloted in the cities of Patna and Mumbai, as well as in Mehsana, a district in Gujarat. The initial designs of the PPIA model was outlined in the NSP for the years 2012–2017 with the technical assistance and funding of the Bill and Melinda Gates Foundation, under the state's RNTCP's new goal of providing “Universal Access to Quality TB Care.” Since the intervention was designed as a pilot, it was modified slightly to track multiple variables for each of these three sites. The PPIA was the first model that aimed to tackle the heterogeneity of the private sector. It aimed to increase notifications, shorten the delay, and increase compliance by policing the private market rather than by excluding it from offering treatment. The PPIA differentiated the various services of the intervention, according to the perceived quality of (and authority to provide) the care offered by the different actors in the private sector. The private sector for the purpose of this intervention was divided into the following:

**Table:** Central TB Division, Directorate General of Health Services, Ministry of Health and Family Welfare (2012), p. 58,59.

1	Primary medical service providers	Stand-alone clinics of practitioners of modern medicine Stand-alone clinics of practitioners of indigenous systems of medicine and homeopathy, pharmacists and less-than-fully qualified providers (which some would call “quacks”)
2	Secondary medical service providers	With specialty services, laboratories, and pharmacies
3	Tertiary medical service providers	With higher specialties and function; some may be medical colleges
4	Implementing agencies	May be NGO/INGO, CSO, CBO, FBO, or other
5	Laboratories	Large, networked (chain) or small
6	Pharmacies	Large, networked (chain) or small

According to this division, the practitioners of modern medicine, or those fully qualified—or for the purpose of this paper, “formal providers”—were afforded the authority to give vouchers for free diagnostic tests to people with suspected TB, but they also could diagnose patients on the basis of the tests or their clinical judgment. They were given the authority to offer vouchers for treatment that would enable the patient to obtain anti-TB medications from any nearby affiliated or engaged pharmacist or chemist, rather than go to the public sector for treatment. Furthermore, the patient could visit the doctor every 15 or 30 days to get another voucher treatment for the next batch of medicines. Every 3 months, the patient would be asked to take a sputum test again to check their progress.

The registering of patients under the PPIA scheme would automatically be registering the patient into the RNTCP by the agency, which in Patna was World Health Partners. To give the vouchers, the providers would have to register the patients for either testing or treatment and the registration would allow the patients' treatment as well as the providers' activity to be tracked. Laboratories were engaged where the vouchers could be redeemed and chemists were also engaged and reimbursed for the anti-TB medications that were sold through vouchers. These chemists were directed to stock the anti-TB medicines that had been approved by the government, which the providers were trained to prescribe^2^. The radical break that the PPIA model advocated was that the private providers would notify patients into the RNTCP, but they did not need to refer them to the public sector for treatment. Rather, they could prescribe treatment that the PPIA agency would ensure followed the Standards for TB Care in India (STCI). Wells et al. write, “Results-based financing has been tried both by funding agencies and by governments for disbursement of their own money, but governments can be reluctant to directly fund private sector entities with domestic resources. Thus, there has been limited work on creating efficient, results-based pathways from governments to private providers” (Wells et al., 2015, p. 6). In other words, governments have been reluctant to reimburse private sector profits with public monies. The Gates Foundation, as a technical advisor to the Government of India in their formulation of the NSP to combat TB, agreed to fund the private sector in their treatment of TB patients. Therefore, the PPIA is singularly placed, at least in India, to inform us about the pattern of private providers' clinical protocol in addressing TB. As the PPIA allowed providers to dispense and oversee treatment that was free for the patients, the act of notification became different from the act of diagnosis, and neither could address the delay in diagnosis. In other words, a provider could possibly not diagnose patients immediately, but later in their subsequent visits, and they might diagnose, but still not notify.

Furthermore, the PPIA had not barred or forbade the doctors from using sputum AFB instead of GeneXpert; the intervention had assumed that since GeneXpert was technically much superior, the providers would obviously gravitate toward its utilization. Since the use of sputum AFB was already very low, the intervention had to encourage microbiological confirmation for diagnosing among formal providers along with the use of GeneXpert. These two objectives were not initially seen as contradictory, since it was reasonably expected that the incentivized behavior change once established—diagnosing on the basis of microbiological confirmation—would migrate from older technology to newer technology^3^. The clinical realities that I describe below, however, transformed this simultaneous encouragement of older technology into a competition with newer technology. Another set of contradiction emerged because the PPIA pilot made GeneXpert available only in the private sector in Patna, even though its use was being scaled up slowly across the country. While GeneXpert seemingly dovetailed the ambitions of the state in bringing the private sector under control, and global health, in providing quick affordable diagnosis, it inevitably ended up competing with other tests including those that check for drug susceptibility.

## The Expert in the Machine

CBNAAT (GeneXpert) was initially developed in 2002 by Cepheid, an American diagnostics company, to rapidly detect anthrax following the panic ensuing in 2001 when anthrax spores were sent through the United States Postal Service. The Foundation for Innovative New Diagnostics (FIND), a Swiss non-profit that funds product development, recognized the potential of GeneXpert for TB control and approached Cepheid in 2006 along with funding from the US National Institute of Health and the Gates Foundation to adapt the technology for TB. David Alland at the University of Medicine and Dentistry of New Jersey, whose research centers on rapid diagnostics for biodefense and antiobiotic resistance, provided technical input to develop cartridges that could be used for TB and hence made possible for GeneXpert to be transferred from the context of bioterrorism to Global Health. The FIND was already a member of the Stop TB Partnership and had a “working relationship” with the World Health Organization (WHO) (n.d.). Alongside this existing network of actors, the pathological mechanism that contributed to the rapid endorsement and uptake of GeneXpert, at least by policy makers and global health organizations, is that instead of testing for antigens or antibodies, it detects genetic material. GeneXpert also bypasses the need to conduct a culture test that detects resistance or sensitivity: it can simultaneously detect the bacterium as well as its sensitivity to rifampicin, the compound in anti-TB medications. Existing tests like the Drug Sensitivity/Susceptibility test (C-DST), which uses liquid culture, remain the gold standard and take over 2 months to give results. The Line Probe Assay is the other test that can check a sputum sample for the presence of bacterium as well as resistance to drugs by amplifying the DNA present by polymerase chain reaction and can take 7–15 days to yield results. Yet, the “technologic imperative” for rapidly testing samples for resistance cannot be said to have been solely driven by drug-resistant TB (Fuchs, 1968); the disease remains notoriously strapped for funding given the unequal distribution of its burden with the poorest neighborhoods suffering the most (Pai, 2018). Nevertheless, after it had been established that drug-resistant TB can be transmitted and not just acquired through intermittent treatment in the early 1990's, existing research and innovation was put to work to make GeneXpert available to global health (Porter and Farmer, 2013).

The lack of quick reliable tests was also keenly felt in Patna. The existing tests (C-DST and Line Probe Assay) were considered very expensive and doctors prescribed them very rarely, claiming that their patients could not afford them. A doctor told me that in addition to the fact that the tests were available in only two labs in the entire city, the samples were not actually tested in Patna but were sent to New Delhi to another lab for testing; thus, the test results would take even longer for patients in Patna. Apart from the constraint of time, the issue of affordability, the market, and class played out in a very specific way. When I enquired why these tests had not become popular in the private sector in Patna, a doctor informed me that if a patient could afford the tests, they would usually travel to another nearby city, like Calcutta, Delhi, or Mumbai, for their treatment. Because of time constraints, along with better facilities available in other places, Patna then never faced pressures from the market to supply these tests. By 2012, it was reported that the public sector was woefully ill prepared to test samples for drug resistance. The NSP stated, “As of December 2011, 35 C-DST labs were accredited under RNTCP to provide services for the diagnosis and follow-up of MDR TB patients [Multi-Drug-Resistant TB]. Eighteen of the 35 accredited C-DST labs are also accredited under RNTCP to provide services for diagnosis of MDR-TB using a rapid molecular test—the Line Probe Assay (LPA) that has a turnaround time of 48–72 h as compared to 3–4 months in conventional testing” (Central TB Division, Directorate General of Health Services, Ministry of Health and Family Welfare, 2012, p. 68). These extenuating circumstances in TB control led to accelerating the assessment of GeneXpert's efficacy and accuracy for diagnosing TB (Boehme et al., 2010; Small and Pai, 2010), and in December of 2010, the WHO endorsed its use by calling it “a major milestone for global TB diagnosis and care” [World Health Organization (WHO), 2010]. Given the issue of missed and late diagnoses because of lack of use of sputum microscopy, use of ineffective tests as mentioned above, and the threat of drug resistance, the importance and hope attached to GeneXpert cannot be overstated. Satyanarayana et al. (2015) reported “considerable heterogeneity in the proportion of providers who were aware that patients with suspected pulmonary TB should undergo sputum examination, ranging from as low as 17% to as high as 94%. Five studies that provided information on practices (mostly by interviewing patients regarding provider practices) reported that, of persons with cough of 2–3 weeks' duration, only 11–59% were advised to undergo sputum examination” (p. 755). To address this lack of use of sputum to diagnose TB patients, the NSP in India guided by the WHO also endorsed the test. The responsibility of demonstrating its feasibility was put on the PPIA pilot intervention.

In her analysis of new technology adoption, Koenig writes, “once a new technology is developed, the forces favoring its adoption and continued use as a standard therapy are formidable” (Koenig, 1988, p. 467). I have attempted to describe the formidable forces working at the level of the pathology of the disease, public health research, and global health organizations and I now turn to how GeneXpert was made available in India. Given the exorbitant cost of the GeneXpert machines and hence the cost of the test, the next immediate goal was to demonstrate the feasibility of large-scale use of GeneXpert, which would also then distribute the cost of such an expensive endeavor. An initiative was launched in 2013 by a coalition of varied stakeholders including 23 private diagnostic companies and the Federation of Indian Chambers of Commerce and Industry, with funding from the Clinton Health Access Initiative, to make GeneXpert available and affordable in India (Kay, 2013). This coalition was called the “Initiative for Promoting Affordable, Quality TB tests” (IPAQT) and it negotiated between the private labs and cepheid for procuring the tests at a cheaper price in exchange for charging the patients less (Pai, 2013). By the time the PPIA began offering its services in 2013, IPAQT had installed machines widely only in some cities, including Mumbai, but not in Patna where ethnographic research was conducted by the author and the editor (Veena Das) as part of the impact evaluation team. Only two labs in Patna had agreed to join IPAQT to bring GeneXpert testing and one was removed from the program in early 2015 for using expired cartridges; the other decided to drop out at the same time because the volume of patients was not high enough in Patna to counter a low margin of profit. Since there was such low uptake of technology among lab owners, the interface agency that was granted the contract to be the PPIA had to innovate at several levels to make GeneXpert available. The interface agency in Patna was World Health Partners and they were given permission to run their own lab, which would conduct the GeneXpert tests. This was not ideal since the goal was to make GeneXpert sustainable as a profit-making product in the market. It was hoped that after a few years, GeneXpert would be popular enough that the agency would no longer need to run its own lab. So, to popularize GeneXpert, the agency, in partnership with Unihealth Lab, installed machines from the beginning of the intervention. Not only were providers given the relevant literature regarding GeneXpert tests, training sessions were regularly held to explain the emerging rifampicin resistance in patients. The infrastructure was further developed to ensure the smooth and seamless use of GeneXpert with technicians trained to run the tests, as well as support made available in case the machines malfunctioned and stopped working.

## Art, Evidence, and Expertise

During my 20 months of fieldwork in Patna, the capital city of the eastern state of Bihar, I conducted research that included interviewing and shadowing formal and informal providers. I also interviewed compounders (doctors' assistants in clinics), lab owners and technicians, pharmacy shop owners and their assistants, patients, as well as the large field staff that manned the PPIA intervention on the ground. I recorded these conversations in an attempt to understand how the PPIA intervention was received and recognized by the various actors that comprised the medical infrastructure and market of the city. The pattern of uptake that emerged showed that rather than GeneXpert simply replacing an older unreliable technology, adjustments needed to be made continuously in how the new technology was offered, addressing concerns that were not only particular to the city but also particular to the practices of providers and patients. In short, Patna was not a passive location where GeneXpert was welcomed without suspicion. The vacuum that the new technology was filling was a scientific construction—the new technology did not represent an articulation of preexisting anticipation or demand^4^. The rich body of work broadly called the “sociology of expectations” has shown how new technology is discursively produced with hope, expectations, and optimism attached to it (Brown and Michael, 2003). Yet, when we look at a different set of experts who are positioned differently to the technology, as in the case of formal providers in Patna, we see suspicion, skepticism, and pessimism. The issue is not one of mistranslation since both sets of experts, the ones who endorse and encourage use of GeneXpert as well as the doctors, one of the consumers of the technology, are trained in the language of science and biomedicine.

To understand the resistance to GeneXpert, we must once again go back to the issue of the low use of sputum AFB to diagnose patients with TB. Since both sputum AFB and GeneXpert located the evidence of TB in the sputum sample of the patient, it was necessary to explore why the providers were not confident of finding the truth of the illness in that instance of pathological markers and technology. The reliability of sputum AFB depends on various factors including the quality of reagent used, the training and experience of lab technicians, but also the sputum sample. The test is most effective if the sputum that is being tested is one that has been expectorated in the morning. These samples have the highest concentration of the bacterium and that cuts the chances of a false negative. Providers complained that since a large share of patients that sought health care in Patna were traveling from the surrounding villages and towns and were unable to return the next day with the sputum sample, samples collected on the spot were often not the best samples to be tested. The patients who lived nearby and were able to return the next day to the doctor or the lab were rarely following the instructions; they would offer excuses that they expectorated their morning sample during brushing and the second sample delivered was not the best for the most accurate results. I must add though, throughout my study, that I never once encountered a doctor explaining the proper way to collect sputum to the patient; the task fell on the compounder who gave vague instructions or the field officers who could only explain but not ensure that the patient would follow through. This disinterest in sputum tests reflected the long mistrust in the labs and the lack of confidence in the ability of the actors to follow instructions rather than a resistance to the established clinical protocol on part of the doctors.

My interview with technicians showed a similar stance toward the high possibilities of the test reflecting inaccurate results but the technicians shifted the blame from patients to the force of market competition. A lab owner said, “Nowadays, a lab technician with even a few months of apprenticeship is borrowing money and setting up shop, he pays the doctors a cut of his earnings and gets patients. He does not have any experience, it took me more than 20 years to become an expert. Books cannot teach you how to recognize that the stain you are seeing is because the reagent is old and has crystallized not because there is a bacterium. You have to learn through experience that if the sputum is too watery you have to heat it so that the water evaporates, but you cannot heat it so much that the sample is destroyed.”^5^ The lab owner was not raising a moot point; the market was perceptibly flooded with small pathology labs that were competing against each other for a share of the patient population. The unregulated diagnostics and pathology labs are constantly a source of complaint since they make effective banning of outdated tests difficult (Jarosławski and Pai, 2012). Furthermore, the experiential and innovative knowledge that plays a role in the quality of services offered were not or perhaps cannot be integrated in the protocols of the intervention. The differential control of these impersonal variables distributed among the patient, provider, and the lab technician resulted in a practice in which providers were reluctant to rely only on one test for confirming a diagnosis or clinical suspicion, and even less on just a sputum sample.

Providers in Patna very rarely diagnosed or tested patients with TB in their first visit and instead ran a therapeutic trial of broad-spectrum antibiotics and other non-specific therapies such as steroids, anti-histaminics, and bronchodilators (McDowell and Pai, 2016). When that failed to provide relief, they would send the patients to be tested for TB. Even then, doctors did not rely on a single test but used a variety of tests to confirm or disprove their clinical judgment. Since the providers were aware of the comorbidities, the history of bodies, and of the local biologies, they relied on a battery of evidence ranging from auscultation to the sound of crackling that lungs with infections produce, X-rays, blood work (to show an infection), sputum microscopy, as well as tests like Mantoux and even banned serological tests. A provider told me that he used a variety of these tests because “there is no one test that can be completely reliable, a patient's breathing might not reveal anything but the X-ray will, if that does not reveal something sputum might, sometimes Mantoux also reveals something.” Each of the top-notifying providers had developed a clinical routine through which they would try and confirm their diagnosis. A gynecologist affirmed her faith in the Mantoux test by irritably saying, “I know it checks only latent TB but sometimes when there is a high load then the induration [pathological marker/bump] will be much larger. I have seen indurations as large as 30 mm.” Providers also said, “Sometimes no test catches it, but I suspect it so I start the treatment therapeutically.” There is good reason to develop a personal clinical routine, not only because there are inevitably variations in the biological and clinical symptoms of TB, but also because the existing infrastructure cannot be entirely trusted to provide reliable results (Achanta et al., 2013). During my fieldwork, the city was also hit with minor medical scandals that uncovered how rejected medical technologies from the First World were being sold in Third World markets at cheaper prices. The diagnostics market, along with the history of patients' and providers' experiences, had implications for the promise that GeneXpert was said to offer.

Through the course of the intervention, it became evident that the private sector operated very differently from city to city, responding to different anxieties and different challenges to their authority. The intervention experimented with allowing informal providers to prescribe GeneXpert on the basis of the expectation that if the patients went to the formal provider with a test result, they would be put on treatment faster—cutting the delay in diagnosis. Formal providers in Patna bristled at this change and threatened to stop participating in the program altogether. Patna was in no shortage of formal providers with affordable expertise and thus there was a smaller number of informal providers filling in the gap for care that is so prevalent in other cities and even more acutely present in rural India. The informal providers were a constant source of irritation and anger for formal providers trained in biomedicine; the latter saw the former as illegally extending their authority when they prescribed scheduled drugs and ordered diagnostic tests, when only formal licensed providers could offer patients such services. Thus, this experiment too had to be halted with a provider complaining that, “if you are giving the informal providers the power to do all this, then what's the need of formal providers?” Since there were not many trained chest specialists in Patna, TB care was handled by general physicians who had gained a reputation through their practice as “TB doctors.” Bombay, unlike Patna, was populated by super-specialized providers who did not have to fight for authority against informal providers. Primary care was neither expected nor given by formal providers in Bombay and, thus, domains of authority and expertise remained more clearly marked. Patna's large population of general physicians had to continually fight with the informal providers to serve as the primary provider for families; they saw giving the informal providers the authority to prescribe a test as empowering “quacks.”

Another effect of the unstable position of formal providers was that providers were careful not to risk their practice being accused as unethical and corrupt. The intervention initially offered GeneXpert for free from May 2014 to March 2015 before experimenting by offering it from April 2015 to May 2016 for a subsidized cost of INR 300 (around 4 USD) for patients who could afford it, while continuing to offer it at no charge for patients below the poverty line. This subsidization proved to be unpopular for several reasons. Since the advertisement at all the networked and officially designated places announced boldly that diagnostic tests were free, doctors reported that patients became suspicious when they were asked to pay INR 300 and accused them of corruption. Doctors further said that patients remained suspicious, even when they were told that sputum AFB was free but not GeneXpert and that their illness required that they be checked for rifampicin resistance. Furthermore, providers reported that patients became irritated when they were told that they had to get their sputum tested again. Providers said, “Patients get angry and ask, “Why are you getting another sputum test done? You had just done it a few days ago?” They think we are making money out of this so they don't want to get it done.” Not wanting to displease patients, the GeneXpert was then not used to diagnose TB initially by doctors but only to diagnose drug resistance cases when they saw and suspected that treatments were failing. The subsidization failed to meet the goal of cheaper and more accurate testing for earlier diagnosis of TB. It could not have been predicted that patients would be more aware than had been assumed regarding the information they were gathering from the various advertisements and social media messages targeted at them.

Joyce (2005), in her work on the use of magnetic resonance imaging (MRI), remarks that experts created and consumed the MRI as being “interchangeable” with the person. MRI, for Joyce's physicians, enabled them to see a reality and truth that patients could not provide; technology offered a transparent knowledge that established a relationship between the physician and the pathology, unhindered by the patient. Providers in Patna did not view GeneXpert as holding any such promise and viewed the intervention's insistence on the use of GeneXpert and the official protocol as threatening. Unlike MRI, GeneXpert was not introduced as being interchangeable with the patient but the strong encouragement of its use made it seem as if it could be interchanged or replace the clinical expertise of the provider in Patna. Several providers told me, “Look, X-ray is not showing anything, sputum is not showing anything, but GeneXpert showed it,” or “You say that GeneXpert is very accurate, but it is not showing anything, but X-ray is clearly showing abnormalities, sputum [microscopy] caught it.” Perhaps the metaphor of catching comes closest to describing how providers utilized diagnostic tests and signals to us providers' perception of their skill, knowledge, and training as art rather than following protocols. Providers in Patna waxed eloquently about their expertise, which they had honed over decades of experience^6^. They were quick to take offense at suggestions that technology could reproduce the art of diagnosing, healing, treating, curing, and caring that they had mastered^7^. Though they did not question the way in which evidence-based medicine was being utilized, in so many words, they did have strong opinions on how new medical devices were flooding the market and being marketed by pharmaceutical representatives in their clinics on a daily basis (Greenhalgh et al., 2014). A provider who had gained a reputation as a TB specialist in Patna welcomed the opportunity to discuss GeneXpert with me and said, “Look, I have read the literature I was provided. The test is not 100 percent accurate. They say so themselves—there is quite a large chance that the results are false. It is like any other machine that these medical representatives come to sell—every week there is a new asthma machine, a new sugar machine, they come and they disappear. How can one rely on them? At the end of the day I will have to rely only on my knowledge when I see the patient.” Another provider who found the insistence on sputum and any kind of diagnostic tests very amusing said, “I have practiced for over 30 years and not in cities but in villages, there was no X-ray machine, lab, and all this *tam-jham* (hassles), and I have diagnosed, treated, and cured hundreds of patients just using my knowledge. I do not need to rely on these tests at all.” Older technology had always been secondary to the providers' judgment and still was in the national plan cited above. But in the intervention's design, incentives and targets for the various actors (including the overworked and underpaid field officers) were tied to not just GeneXpert utilization but also positive diagnosis yielded by that new piece of technology. The emphasis on GeneXpert in the intervention design thereby set up a confrontation between global health technology and providers and created a new point of tension between expertise and technology. Providers resented this call and eventually GeneXpert had to be utilized and weighted differently given the less than enthusiastic reception it received.

## Tried, Tested, Trusted, and Failed: Concluding Comments

GeneXpert is a nimble technology that can be adapted to different diseases; different cartridges can be used to adapt the machine to different diseases, such as hepatitis, HIV, chlamydia, and gonorrhea. Cazabon et al. (2018) show that the machine is being employed in different countries across the world for different diseases and being underutilized for TB. These results reveal a certain difference in how urgency and crisis are perceived across various sites: global, national, city, and even neighborhood. This is made clearer when we look at how GeneXpert failed to live up to its promises for a different epidemic, namely, the Ebola outbreak of 2014–2016. The context of nearly absent primary health care diagnostic facilities in Sierra Leone resulted in a lack of infrastructure that would have supported GeneXpert uptake, hence ensuring the low utilization of GeneXpert (Vernooij, 2019). The experimentation with the introduction and use of GeneXpert reveals the various competing ontologies of disease. Hence, while technology both older and newer reveals a relationship that is focused on the pathogen, clinical practice reveals another that is fixed on the person. Relatedly, the different ways in which the various actors define the urgency of the disease further reveals that the temporality of the epidemic is not always the temporality of the patient's illness.

The concerns of global health with the urgencies and transformation of the epidemic were not how the providers were encountering their patients. The concerns of global health with the urgency and transformation of the epidemic were not reflected in how the providers were encountering and treating their patients. The discourse around “the missing million” and “disease eradication” never emerged in the interviews with the practitioners in the private sector. The failure to follow the protocol cannot be attributed to a lack of training, motive, or profit, since all three were present and accounted for with the formal providers. The unwillingness to relinquish expertise to technology mirrors, in some ways, the earlier unwillingness of providers to relinquish patients' treatment to the public sector. Providers in Patna in their complaints about how the implementation of GeneXpert was threatening their expertise were illustrating what Webster in this volume has argued, that the “language of acceleration and faster delivery” be interrupted by reaching an agreement on how complexity be handled (Webster, 2019, p. 4). The complexity that the providers in Patna were pointing toward was one of unpredictable variation in how technology and biology intersected. TB could only be recognized both, through and despite diagnostic technologies. This instrumental relationship with technology worked against GeneXpert's reception as a magic bullet; in short, providers were not so much as against using technology but against it replacing expertise. It was precisely because providers were well-aware of the complexity of TB—in the unexpected ways it afflicts and reveals itself in a patient—that they were unwilling to accelerate use of GeneXpert at the cost of their own skill, expertise, and experience.

Hence, to conclude, to some extent, the story of GeneXpert is a familiar story, even within the narrow scope of TB diagnostics. As Small and Pai remind us, the conventional nucleic acid amplification tests “have been licensed for nearly 20 years and yet have not had a substantial effect on tuberculosis control” (Small and Pai, 2010, p. 1071). Studying experts in places that are usually the sites of global health intervention reveal a limit to the emphasis on hope and hype that is embedded in new genetic technology (Rose and Novas, 2005). In contrast, the affects tethered to GeneXpert were skepticism and doubts of private providers. Hence, rather than technological innovation moving in a unilinear direction (upstream or downstream), the local professional and social context within which it is interpreted and used in practice has vital implications for what the future of global public health might hold, not only for TB but also for new health technologies in general (Street, 2018). This paper has tried to highlight how such shifts in India necessarily come up against the knotty relation between the state, the market, and private providers, as seen in Patna.

## Data Availability Statement

The datasets generated for this study are available on request to the corresponding author.

## Ethics Statement

The studies involving human participants were reviewed and approved by Institute of Socio-Economic Research on Development and Democracy (ISERDD), New Delhi, India. Written informed consent for participation was not required for this study in accordance with the national legislation and the institutional requirements.

## Author Contributions

The author confirms being the sole contributor of this work and has approved it for publication.

### Conflict of Interest

The author declares that the research was conducted in the absence of any commercial or financial relationships that could be construed as a potential conflict of interest. The reviewer MP declared a past co-authorship with the author VS to the handling editor.

## Footnotes

^1^I wish to acknowledge the comments of Veena Das, Jishnu Das, Madhukar Pai, Ada Kwan, Ben Daniels, and the entire Qutub Project team, especially ISERDD that made my research possible and the article richer.

^2^The PPIA can be seen to modify previous models to engage the private sector to the extent that the providers were given the authority to initiate and oversee treatment, albeit under the supervision of the PPIA agency. The PPIA was tasked with the responsibility of controlling the regimen that could be prescribed, thereby making sure the patients that did get registered were being treated according to the protocol set by WHO and publicized as the STCI.

^3^Initially, there were other concerns as well, which the intervention had to continuously tinker with as more and more data were collected; for example, since GeneXpert is relatively more expensive than sputum AFB, the PPIA had no existing data on how providers would convince patients to bear the costs initially. Given the additional value that GeneXpert brought to the clinic, and that the test was made affordable through various market logics, it was expected that the comparatively more expensive GeneXpert would replace the cheaper and unreliable Sputum AFB. This was not the case and very quickly the intervention corrected itself to make GeneXpert completely free just like the sputum AFB, but this was not what set up a competition between the older and newer technology.

^4^It must also be mentioned that this was specific to the landscape of Patna; other cities where the multiple actors have a different relation to each other have resulted in a different pattern on how GX is used. For example, see McDowell et al. (2018) for how GX was utilized by informal providers in Mumbai.

^5^All participants were informed as to the purpose of the interview, which was recorded with their consent. This consent included consent for publication of their verbatim quotes.

^6^The narratives of professional accomplishment and skill that providers gave me were tied to the moral and ethical self-fashioning that they claimed to have achieved. Since a large number of these providers had worked in the public sector in the past with far less resources, their work and accomplishments were seen in service to the nation. Hence, I use the word “authority,” but perhaps “sovereignty” is a better descriptor of their affect since their work, action, and authority was tied to the sovereignty of the Indian nation state. I thank Stefan Ecks for this point who has written on this issue (Ecks, 2004, 2013).

^7^For another example of how different cities with their own particular arrangement of actors and network produce different reactions to GeneXpert, please see McDowell et al. (2018).
